# Diffusion model comparison identifies distinct tumor sub‐regions and tracks treatment response

**DOI:** 10.1002/mrm.28196

**Published:** 2020-02-14

**Authors:** Damien J. McHugh, Grazyna Lipowska‐Bhalla, Muhammad Babur, Yvonne Watson, Isabel Peset, Hitesh B. Mistry, Penny L. Hubbard Cristinacce, Josephine H. Naish, Jamie Honeychurch, Kaye J. Williams, James P. B. O'Connor, Geoffrey J. M. Parker

**Affiliations:** ^1^ Quantitative Biomedical Imaging Laboratory The University of Manchester Manchester UK; ^2^ Division of Cancer Sciences The University of Manchester Manchester UK; ^3^ Division of Pharmacy & Optometry The University of Manchester Manchester UK; ^4^ Imaging and Flow Cytometry Cancer Research UK Manchester Institute Manchester UK; ^5^ Division of Cardiovascular Sciences The University of Manchester Manchester UK; ^6^ Bioxydyn Ltd. Manchester UK; ^7^ Division of Neuroscience and Experimental Psychology The University of Manchester Manchester UK; ^8^ Centre for Medical Image Computing University College London London UK

**Keywords:** diffusion‐weighted MRI, microstructural model, model selection, necrosis, radiotherapy, tumor microstructure

## Abstract

**Purpose:**

MRI biomarkers of tumor response to treatment are typically obtained from parameters derived from a model applied to pre‐treatment and post‐treatment data. However, as tumors are spatially and temporally heterogeneous, different models may be necessary in different tumor regions, and model suitability may change over time. This work evaluates how the suitability of two diffusion‐weighted (DW) MRI models varies spatially within tumors at the voxel level and in response to radiotherapy, potentially allowing inference of qualitatively different tumor microenvironments.

**Methods:**

DW‐MRI data were acquired in CT26 subcutaneous allografts before and after radiotherapy. Restricted and time‐independent diffusion models were compared, with regions well‐described by the former hypothesized to reflect cellular tissue, and those well‐described by the latter expected to reflect necrosis or oedema. Technical and biological validation of the percentage of tissue described by the restricted diffusion microstructural model (termed %MM) was performed through simulations and histological comparison.

**Results:**

Spatial and radiotherapy‐related variation in model suitability was observed. %MM decreased from a mean of 64% at baseline to 44% 6 days post‐radiotherapy in the treated group. %MM correlated negatively with the percentage of necrosis from histology, but overestimated it due to noise. Within MM regions, microstructural parameters were sensitive to radiotherapy‐induced changes.

**Conclusions:**

There is spatial and radiotherapy‐related variation in different models’ suitability for describing diffusion in tumor tissue, suggesting the presence of different and changing tumor sub‐regions. The biological and technical validation of the proposed %MM cancer imaging biomarker suggests it correlates with, but overestimates, the percentage of necrosis.

## 1 INTRODUCTION

Quantitative MR biomarkers can provide a non‐invasive assessment of tumor response to treatment, potentially serving as useful tools in the development of novel therapies, or enabling tracking of patient response and guiding clinical decisions regarding therapy options.^1^, ^2^ In order to fulfil this potential and become robust tools in research or clinical settings, biomarkers require both technical validation, for example, evaluating their accuracy and precision, and biological validation, to understand their relationship to biological processes.^2^

An important consideration in assessing biomarker accuracy and precision is the validity of the model from which the biomarker is obtained. Typically, MRI biomarkers of tumor response to treatment are obtained by calculating changes in summary statistics of parameters derived from a model that is applied to pre‐treatment and post‐treatment data. However, tissue within tumors is known to be heterogeneous,^3^, ^4^ with this intra‐tumor heterogeneity potentially varying over the course of treatment. Single summary statistics, such as parameter means or medians, do not capture such heterogeneity, and, moreover, do not provide information about the suitability of the applied model. For example, different models may be necessary in different regions, and model suitability may change over time, confounding the interpretation of biomarkers obtained from a single model applied to all tumor voxels at all time points. Understanding the applicability of different models may provide information about qualitative differences in the structure or function of tumor sub‐regions, and may enhance the utility of model parameters themselves, for example, by allowing the rejection of values in regions where the model is not appropriate. Such analyses also have the potential to yield new biomarkers based on the classification of tissue according to model suitability.

Model comparison techniques have been applied to a number of models used to obtain biomarkers from quantitative MRI data. For example, model selection in dynamic contrast‐enhanced MRI^5^ has been used to evaluate the suitability of different models for describing average whole‐tumor signal time courses in cervical tumors,^6^ and voxel‐wise model comparison has shown that different models tend to be favored in liver metastases compared with the surrounding liver.^7^ In diffusion‐weighted (DW) MRI, a technique which has seen extensive use in evaluating treatment response,^8^, ^9^ model comparison has shown that non‐monoexponential representations (intra‐voxel incoherent motion (IVIM), the stretched exponential, and the statistical model) tend to be preferred over the monoexponential apparent diffusion coefficient (ADC) before and after androgen deprivation therapy in patients with bone metastases from prostate cancer.^10^ Model comparison has also been used to show that a microstructural model comprising restricted intracellular diffusion, hindered extracellular diffusion and intravascular pseudo‐diffusion describes whole‐tumor DW‐MRI data better than ADC or IVIM, in two untreated models of colorectal cancer.^11^ Recently, a comparison between a microstructural model and ADC has been used to distinguish viable tissue in gliomas from necrotic or oedematous regions, and from surrounding brain tissue.^12^

This work evaluates how the relative suitability of two DW‐MRI models varies spatially within tumors at the voxel level and in response to radiotherapy, potentially allowing inference of qualitatively different tumor microenvironments. Models of restricted and time‐independent diffusion were compared, with regions well‐described by the former hypothesized to reflect cellular tissue, and those well‐described by the latter expected to reflect necrotic, cystic, or oedematous regions. Biological and technical validation of this methodology was performed using in vivo experiments and simulations.^13^

## 2 METHODS

### 2.1 Mice and cell lines

Animal experiments were approved by a local ethics committee and performed under a United Kingdom Home Office license, in compliance with UK National Cancer Research Institute guidelines for the welfare of animals in cancer research,^14^ and with the ARRIVE (Animals in Research: Reporting In Vivo Experiments) guidelines.^15^ All experiments were performed with a syngeneic mouse model, where CT26 murine colon carcinoma cells were implanted in an immunocompetent BALB/c mouse host. Mice were obtained from Harlan (Bicester, UK), and were housed under specific pathogen‐free conditions in individually ventilated cages holding a maximum of 6 animals, with appropriate bedding, nesting material, and a cardboard tunnel. Mice were housed on a 12 h/12 h light/dark cycle and were given filtered water and fed an appropriate rodent diet. CT26 cells (ATCC) were maintained in Dulbecco's modified eagle medium (DMEM), supplemented with 10% fetal calf serum (FCS) and 1% L‐glutamine (Invitrogen), and cultured to limited passage for 1‐2 weeks prior to implantation, with regular re‐screening for mycoplasma contamination. Mice were inoculated subcutaneously in the supraspinal position with $1\;\times \;10^{6}$ CT26 cells in 100 μL of phosphate‐buffered saline, and were treated when tumors were 250‐300 ${\text{mm}}^{3}$, as measured with callipers.

### 2.2 Tumor radiotherapy and MR scan schedule

Mice received either sham therapy (control group, C; *n* = 10), or a single dose of 10 Gy delivered bilaterally (radiotherapy group, RT; *n* = 9). While formal sample size calculations were not performed, these group sizes are similar to those used previously to detect significant cohort‐level changes in ADC.^16^ MR scanning was performed ∼2‐4 hours before sham/treatment (day 0) and at up to three post‐treatment time points (days 3, 6, and 10). Specifically, 3 animals (2 C, 1 RT) were scanned at days 0 and 3; 7 (4 C, 3 RT) at days 0, 3, and 6; and 9 (4 C, 5 RT) at days 0, 3, 6, and 10. These time points were chosen based on previous observations of CT26 tumor growth inhibition and size reductions in response to 10 Gy radiotherapy.^17^ Animals were randomized to control and treatment groups following the day 0 scan. The timing of control and treated scans was not formally randomized, but animals from both groups had scans distributed throughout the morning and early afternoon.

### 2.3 Histology

Animals were euthanized immediately after their last scan, allowing tumors at a range of time points to be harvested for histological analysis. Tumors were excised whole and bisected along the imaging plane, taking for histology the half of the tumor which was closest to the body. These halves were then fixed in 4% neutral buffered formalin for 24 hours, transferred to 70% ethanol, processed and then embedded in paraffin. Sections 5 μm thick were cut, floated out on a water bath, collected on charged slides and then dried at $37^{\circ }$C overnight. Sections were stained with hematoxylin and eosin (H&E) to allow identification of viable and necrotic tumor, and whole‐field images were obtained using a SCN 400 Leica scanner at 40× magnification. Tumors were segmented semi‐automatically into viable and necrotic tissue, and the percentage area of necrosis, % necrosis, was calculated on a single H&E slice for each tumor; this single slice came from the cut face of the tumor half, which approximately corresponds to the tumor center. Pathology image analysis was performed using the Composer module in Tissue Studio Portal version 4.4, Definiens Developer XD version 2.7 (Definiens AG, Munich, Germany).

### 2.4 MR protocol

All scans were performed on a 7 T horizontal bore magnet (Magnex Scientific Ltd., Abingdon, UK) interfaced to a Bruker Avance III console running ParaVision 6.0.1 (Bruker BioSpin, Ettlingen, Germany). All data were acquired using a transmit‐only volume coil for excitation, with a receive‐only surface coil placed over the tumor, with animals in the prone position. Anaesthesia was induced, and was maintained throughout scanning using 2% isoflurane in oxygen, delivered at 2 L/minutes; respiratory rate and core body temperature were monitored, with temperature maintained at $37^{\circ }$C using warm air.

A $T_{2}$‐weighted rapid acquisition with relaxation enhancement sequence was performed for tumor localization, and for subsequent region of interest (ROI) definition; effective TE = 33 ms, TR = 2500 ms, matrix = 256 × 256. For conventional ADC mapping, pulsed gradient spin echo (PGSE) data were acquired with *δ* = 4.65 ms, Δ = 9.86 ms, *G* = 113, 207, 293 mT/m, $b\;=\;150,\;500,\;1000\;s/{\mathrm{mm}}^{2}$, TE = 20.4 ms, TR = 2550 ms, matrix = 128 × 128; this is referred to as the *single diffusion time* dataset. For microstructural modelling and model comparison, PGSE data were acquired with *δ* = 4.65 ms, Δ = 9.86, 40.0 ms, *G* = 0, 113, 207, 293 mT/m, *b* = 0, 150, 500, 1000, $0,\;689,\;2296,\;4592\;s/{\mathrm{mm}}^{2}$, TE = 50.1 ms, TR = 2550 ms, matrix = 64 × 64; this is referred to as the *two diffusion time* dataset. Note that gradient duration, gradient strength, TE, and TR were the same for both diffusion times in the *two diffusion time* dataset. The gradient rise time was 0.245 ms for all PGSE scans, and imaging volumes were identical for all scans, providing full tumor coverage with field of view  = 32 mm × 32 mm, slice thickness  = 0.6 mm, and 20 coronal slices.

### 2.5 MR analysis

For conventional ADC mapping using the *single diffusion time* dataset, voxel‐wise signals, *S*, were normalized to the $b\;=\;150\;s/{\mathrm{mm}}^{2}$ signal to minimize the potential influence of capillary blood flow, and fitted to $S/S_{b150}\;=\;\exp\left(-b\text{ADC}\right)$. For microstructural modelling and model comparison, two models were separately fitted to the *two diffusion time* dataset. First, a two‐compartment microstructural model (MM) of diffusion restricted within impermeable spheres and hindered in the extracellular space^18^ was fitted to signals normalized to *G* = 0 mT/m, estimating cell radius, *R*, intra‐cellular and extra‐cellular diffusivities, $D_{i}$ and $D_{e}$, and intracellular signal fraction, $f_{i}$. Second, a monoexponential decay with *b*‐value was fitted to the same data, yielding a single diffusivity, here referred to as $D^{\prime }$. As data from two diffusion times were included in this fit, the monoexponential decay in this case is only appropriate where diffusion is time‐independent, with the signal depending only on *b*‐value; this is referred to as the time‐independent diffusion (TID) model. Note that this differs from the conventional ADC mapping described above, which only uses a single diffusion time. Potential noise bias was mitigated by discarding signals lower than $2S_{\mathrm{noise}}$, where $S_{\mathrm{noise}}$ is the mean signal in a noise ROI,^19^ and each voxel‐wise fit was performed for 100 starting values, with the final parameter estimates taken as those resulting in the lowest value of the objective function. Diffusion gradient rise times were included in all models,^20^ and parameters were constrained to be within plausible limits: 0.1 ≤ *R* ≤ 25 μm, $0.1\;\leq \;\text{ADC},\;D_{i},\;D_{e},\;D^{\prime }\;\leq \;3\;\mu m^{2}$/ms, $0.01\;\leq \;f_{i}\;\leq \;1$. All analyses were carried out in MATLAB 2017a (The MathWorks, Inc., Natick, Massachusetts), with least squares fitting performed using a Nelder‐Mead simplex algorithm (*fminsearchbnd* in MATLAB).

In addition to the method of fitting the MM described above, a second approach was investigated as a means of improving fit stability. As a compromise between the direct fitting of $D_{i}$ described above, and the approach taken elsewhere of fixing diffusivities to single a priori values,^11^, ^21^ fitting was repeated effectively using a look‐up table for $D_{i}$. Specifically, in separate fits $D_{i}$ was fixed to five different values, $D_{i}\;=\;0.5,\;1.0,\;1.5,\;2.0,\;2.5\;\mu m^{2}$/ms; these five fits were then compared, with voxel‐wise parameter values taken from the fit with the highest $R^{2}$ (see Supporting Information Figure S1). This resulted in $D_{i}$ maps which were discretized (voxels were one of five possible values), while *R*, $f_{i}$, and $D_{e}$ were continuous; all four parameters could vary spatially. The original method and this second approach are referred to as *fit*‐ $D_{i}$ and *discrete*‐ $D_{i}$, respectively.

Both approaches were investigated in simulations (see Section 2.7), with MM fits for *fit*‐ $D_{i}$ and *discrete*‐ $D_{i}$ compared to evaluate the effects on fit stability of fixing $D_{i}$. This evaluation considered the extent to which fits returned parameters with extreme values, taken as at least one parameter being within 1% of the fit constraints. On the basis of this evaluation, the preferred approach (*fit*‐ $D_{i}$ or *discrete*‐ $D_{i}$) was chosen for subsequent analysis.

**Figure 1:**
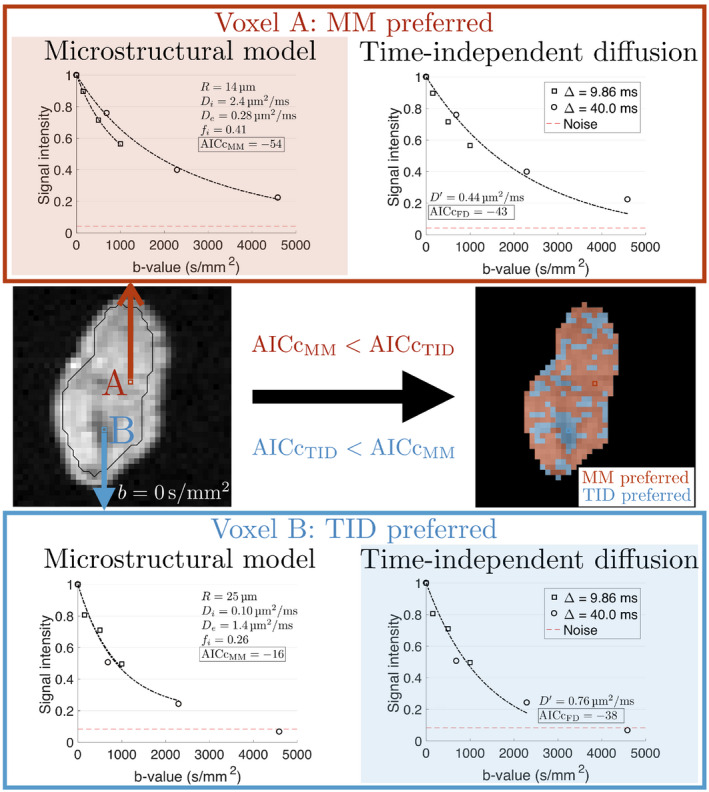
Model selection procedure. The microstructural model (MM) and time‐independent diffusion (TID) model were fitted voxel‐wise to signals normalized to $b\;=\;0\;s/{\mathrm{mm}}^{2}$, with the model resulting in the lowest AICc taken as the preferred model for that voxel. Fits for two example voxels, A and B (red and blue boxes on $b\;=\;0\;s/{\mathrm{mm}}^{2}$ image, respectively) within the tumor ROI (black outline) are shown, with the red dashed line in the plots representing noise floors below which signals are excluded from the fits. The MM model was preferred in voxel A, and TID preferred in voxel B. All voxels in the ROI were then colour coded according to the preferred model, yielding model preference maps (right)

MM fits from the chosen approach were then compared with TID fits on a voxel‐wise basis using the corrected Akaike Information Criterion (AICc), taking the fit with the lower AICc as the preferred model in a given voxel (Figure 1). In AICc calculations, the MM model had four fitted parameters, while TID had one. Within whole‐tumor ROIs, the percentage of voxels with ${\text{AICc}}_{\mathrm{MM}}\;<\;{\text{AICc}}_{\mathrm{TID}}$ was calculated to assess the proportion of tumor tissue in which MM was preferred; this is referred to as %MM. To test the hypothesis that regions well‐described by MM reflect cellular tissue while those well‐described by TID reflect non‐viable tissue, %MM from subjects’ final scan was compared with % necrosis obtained from histology (see Section 2.3). Only %MM data from the central slice of the tumor was used for this correlation, as this corresponded approximately to the region used for histology (see Section 2.3). The link between conventional ADC and histology was also investigated, again only using data from the central slice of the tumor, firstly by comparing median ADC with % necrosis, and secondly by using an ADC threshold to classify necrotic and non‐necrotic voxels. Here, a range of ADC thresholds were applied to central‐slice ADC datasets from subjects’ final scan, in each case calculating the percentage of voxels with ADC below the given threshold, potentially providing a metric analogous to %MM. For each threshold, this metric was compared with % necrosis, to determine an ${\text{ADC}}_{\mathrm{cut}-\mathrm{off}}$ which yields the strongest correlation. All MR image analysis and histology image analysis were performed independently, with each analysis blinded to the results of the other.

MM parameter distributions were then obtained from voxels where MM was preferred, after excluding fits with extreme values (where at least one parameter was within 1% of the fit constraints). Distributions of conventional ADC measurements were obtained from all voxels in a ROI.

### 2.6 Statistical analysis

Median values from distributions for ADC, *R*, $D_{e}$, and $f_{i}$, along with values for %MM, were analysed in a mixed‐effects model, with scan day as a fixed effect, and subject as a random effect. Parameters were modelled as a quadratic function of scan day, to capture the non‐linear trends observed for most parameters. Two models, one without and one with a group/time interaction (with group referring to control or treated animals, and time referring to scan day), were fitted and compared using a likelihood‐ratio test. This procedure was performed for each parameter, with *P* < .05 in the likelihood‐ratio test taken to indicate a statistically significant difference between groups for a given parameter. Mixed‐effects modelling was carried out in R version 3.5.1^22^ using the *nlme* package.^23^

### 2.7 Fitting simulations

Simulations were used to investigate the *discrete*‐ $D_{i}$ approach described above (see Section 2.5). Simulated MM signals were generated for 96 different microstructures (all combinations of *R* = 5, 10 μm, $D_{i},\;D_{e}\;=\;0.2,\;1.1,\;2.0,\;2.9\;\mu m^{2}$/ms, $f_{i}\;=\;0.25,\;0.50,\;0.75$), using the experimental acquisition protocol (see Section 2.4). One thousand and five hundred noisy synthetic signals were generated for each microstructure, with noise added such that the signals were Rician distributed with a signal‐to‐noise ratio (SNR) at $b\;=\;0\;s/{\mathrm{mm}}^{2}$ of 54, matching the mean SNR in the experimental data; SNR was calculated by dividing $b\;=\;0\;s/{\mathrm{mm}}^{2}$ signals by the Rician noise standard deviation, derived from the mean signal in a background ROI.^24^ MM was then fit to the signals using the *fit*‐ $D_{i}$ and *discrete*‐ $D_{i}$ approaches, following the fitting procedure described above (see Section 2.5). The accuracy of model parameter estimates was then evaluated, along with the extent to which the two fitting approaches yielded estimates with extreme values, taken as at least one parameter being within 1% of the fit constraints.

### 2.8 %MM simulations

Simulations were also performed to evaluate the accuracy and precision of %MM measurements. DW signals were simulated from MM and TID models using the experimental acquisition protocol, generating different ‘synthetic tumor datasets’ with ground truth %MM values from 10% to 90%. For each ground truth, 2000 signals were generated using model parameters sampled at random from those obtained in the experimental data; these signals were then split into groups of 200, giving 10 datasets for each ground truth. Noiseless and noisy datasets were generated, with the latter reflecting the SNR properties of the experimental data. All datasets were then analysed with the same pipeline used for the experimental data, that is, fitting MM and TID models, and then performing the AICc analysis. %MM bias was evaluated by comparing calculated values for each dataset with the ground truth, and precision was evaluated by assessing the variability over individual datasets. As a binary classification underlies the calculation of %MM, standard summary statistics of accuracy, sensitivity, and specificity were derived from the confusion matrix to evaluate the technical performance of %MM measurements.^25^ Analysis code will be made available at https://gitlab.com/manchester_qbi/manchester_qbi_public/diffusion_model_comparison.

## 3 RESULTS

### 3.1 Fitting simulations

**Figure 2:**
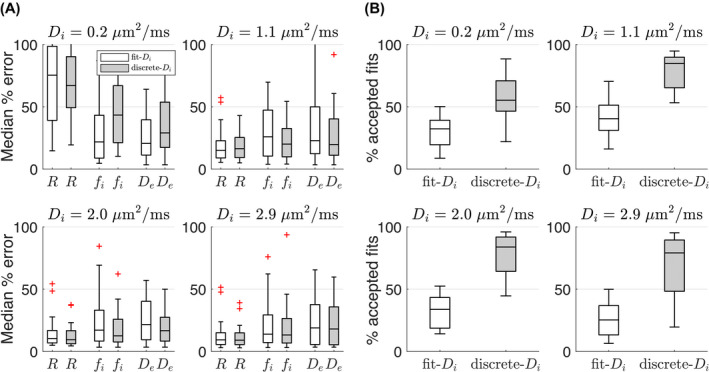
Fitting simulations. A, Boxplots of median percentage error in model parameters for *fit*‐ $D_{i}$ (white) and *discrete*‐ $D_{i}$ (gray) fitting, for four ground truth $D_{i}$ values. B, Boxplots of the percentage of accepted fits (ie, where no parameter is within 1% of the fit constraints), for *fit*‐ $D_{i}$ (white) and *discrete*‐ $D_{i}$ (gray) fitting, for four ground truth $D_{i}$ values. In A and B boxplots in each panel represent distributions over the 24 different ground truth microstructures for the given ground truth $D_{i}$, together presenting results for all 96 microstructures generated

Figure 2A compares the accuracy of model parameter estimates from *fit*‐ $D_{i}$ and *discrete*‐ $D_{i}$ fits, for four ground truth $D_{i}$ values. The accuracy metric was taken as the median absolute percentage difference between each fit result and the ground truth, with the boxplots in each panel representing the distribution over 24 ground truth microstructures with different *R*, $D_{e}$, and $f_{i}$, for the given ground truth $D_{i}$; taken together, the plots present results for all 96 microstructures generated. The *discrete*‐ $D_{i}$ fits tend to have slightly narrower distributions, with fewer large errors, except for $D_{i}\;=\;0.2\;\mu m^{2}$/ms where *discrete*‐ $D_{i}$ would be expected to perform poorly as $D_{i}$ here cannot be lower than 0.5 $\mu m^{2}$/ms (due to the discretization). Although there is not a dramatic improvement in accuracy with *discrete*‐ $D_{i}$, it does tend to result in fewer fits with extreme values, as shown in Figure 2B, where the boxplots show the percentage of accepted fits (that is, where no parameter is within 1% of the fit constraints), as a distribution over 24 microstructures for four ground truth $D_{i}$ values. It should be emphasized that there is a wide variation in parameter accuracy and precision, depending on the ground truth microstructure, and that $D_{i}$ in general tends to be estimated poorly (see Supporting Information Figure S2). Given the improvement in fit stability suggested by Figure 2B, *discrete*‐ $D_{i}$ was used for the in vivo analysis.

### 3.2 %MM simulations

**Figure 3:**
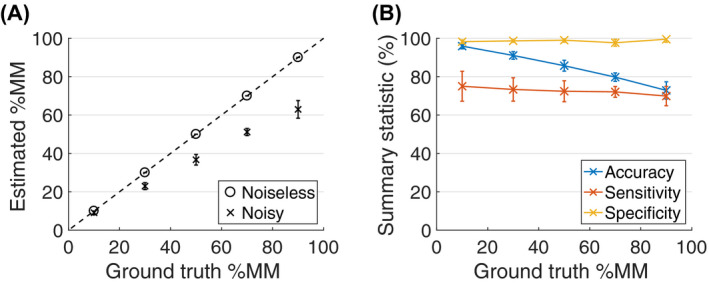
%MM technical validation simulations. A, Estimated %MM values (mean ± standard deviation) plotted against ground truth, for noiseless (circles) and noisy (crosses) datasets. The dashed line is the line of identity. B, Accuracy, sensitivity, and specificity (mean ± standard deviation) plotted against ground truth, for the noisy datasets

Figure 3A plots estimated %MM values (mean ± standard deviation (SD) over datasets) as a function of the ground truth, for noiseless and noisy cases. The infinite SNR case performs as expected, with estimated values matching the ground truth. With noisy signals, however, %MM tends to be underestimated, with the magnitude of the bias increasing with the ground truth value. The degree of underestimation suggests that, for this SNR, estimated %MM values cannot exceed ∼70%, even if the ground truth is higher. Coefficients of variation for %MM are <10% across the ground truth values, indicating good precision, and an insensitivity to different model parameters and noise instances. For the noisy data, Figure 3B plots summary statistics of the classification's confusion matrix, showing high specificity (≥95%), but lower sensitivity (≥60%), with accuracy dropping from a mean of 96% to 73% as ground truth %MM increases from 10% to 90%.

### 3.3 Conventional ADC

**Figure 4:**
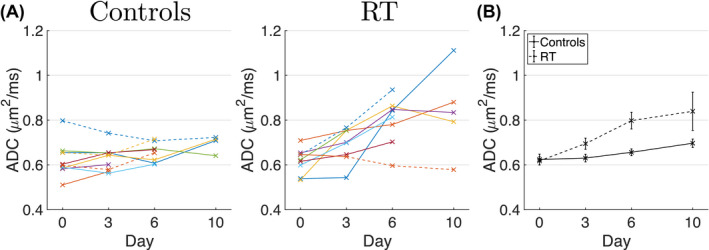
Conventional ADC over time. A, Whole‐tumor median ADC values plotted as a function of time, for control (left) and radiotherapy (RT, right) groups. Individual data points are median values for a given tumor, and lines connect the same tumor at different time points. B, Same data as (A), plotted as mean ± standard error over subjects, for control (solid line) and treated (dashed line) groups

Figure 4A plots median conventional ADC values for all tumors at all time points, with group‐level trends compared in Figure 4B. Note that these conventional ADC values were obtained from whole‐tumor ROIs, irrespective of MM and TID fits. Significant differences in ADC were observed between groups (*P* < .0001), with day 0 and day 10 values (mean ± SD) of $0.62\;\pm \;0.08\;\mu m^{2}$/ms and $0.70\;\pm \;0.04\;\mu m^{2}$/ms for controls, and $0.62\;\pm \;0.06\;\mu m^{2}$/ms and $0.8\;\pm \;0.2\;\mu m^{2}$/ms for treated. Median ADC from subjects’ final scan showed a positive correlation with % necrosis determined from histology (Pearson's correlation coefficient, *ρ* = 0.56, 95% confidence interval (CI) = 0.13 to 0.81, *P* = .016; see Supporting Information Figure S3). An ${\text{ADC}}_{\mathrm{cut}-\mathrm{off}}$ of $1.07\;\mu m^{2}$/ms provided the strongest negative correlation between the percentage of voxels below that value and % necrosis (Pearson's correlation coefficient, *ρ* = −0.65, 95% CI = −0.86 to −0.26, *P* = .003; see Supporting Information Figure S4).

### 3.4 Diffusion model comparison

#### 3.4.1 Model preference relates to histology measurement of necrosis

**Figure 5:**
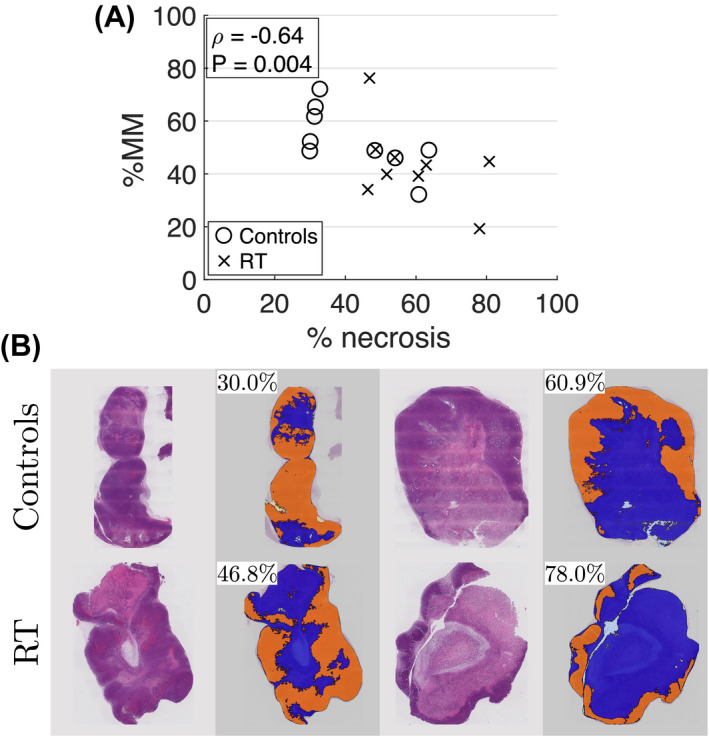
%MM and % necrosis. A, %MM plotted against percentage necrosis for control (circles) and radiotherapy (RT, crosses) groups. Using all data points, there is a significant negative correlation (Pearson's correlation coefficient, *ρ* = −0.64, *P* = .004). B, Example H&E images for two control and two RT tumors, illustrating the staining and segmentation used to determine % necrosis. Orange and blue regions in the segmentation correspond to tumor and necrosis, respectively

Both MM‐favored and TID‐favored voxels were observed in all tumors at all time points, with %MM values ranging from 29% to 76%. At day 0, mean ± SD (over all tumors) was 61 ± 7%. Figure 5A plots %MM against percentage necrosis determined from histology. As with the ADC correlation described above, the %MM values come from subjects’ final scan, that is, the time point closest to the histological analysis. Using data from control and treated tumors, there is a significant negative correlation between %MM and % necrosis (Pearson's correlation coefficient, *ρ* = −0.64, 95% CI = −0.85 to −0.24, *P* = .004). Figure 5B shows example H&E images from control and treated tumors with low and high levels of necrosis, illustrating the staining and segmentation used to determine % necrosis.

#### 3.4.2 Model preference is sensitive to radiotherapy‐induced changes

**Figure 6:**
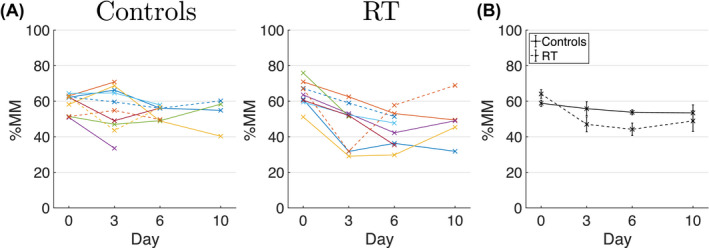
%MM over time. A, Percentage of voxels where the microstructural model (MM) was favored over TID, plotted as a function of time for control (left) and radiotherapy (RT, right) groups. Individual data points are values for a given tumor, and lines connect the same tumor at different time points. B, Same data as (A), plotted as mean ± standard error over subjects, for control (solid line) and treated (dashed line) groups

Figure 6 plots %MM as a function of time, showing a significant difference between control and treated groups (*P* = .0014). %MM decreased from a mean of 64% at baseline to 44% 6 days post‐radiotherapy in the treated group, with 3 out of 5 tumors then showing an increase from day 6 to day 10. These were the same three tumors that showed a decrease in ADC from day 6 to 10 (Figure 4A). Over the same time period, %MM in the control group decreased from a mean of 59% to 54%.

#### 3.4.3 Microstructural parameters exhibit spatial heterogeneity

**Figure 7:**
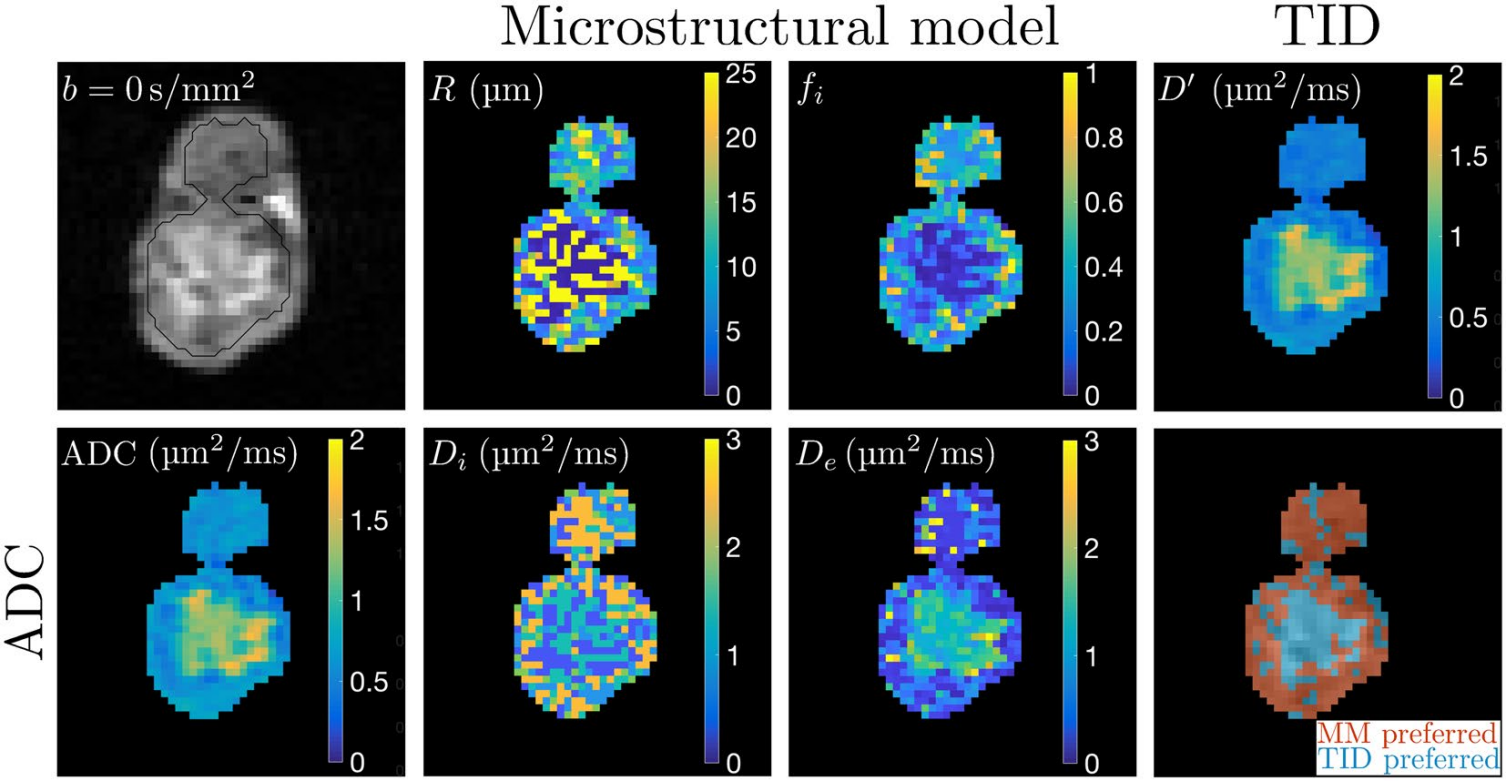
Example $b\;=\;0\;s/{\mathrm{mm}}^{2}$ image at day 6 (top left, with ROI in black) and corresponding conventional ADC map (bottom left). Parameter maps for MM (central four panels) and TID (top right) are also shown, along with the model preference map (bottom right, overlaid on $b\;=\;0\;s/{\mathrm{mm}}^{2}$ image for voxels in the ROI). The TID model was preferred in the central region of high $b\;=\;0\;s/{\mathrm{mm}}^{2}$ image signal, while MM tended to be preferred around the rim and throughout the smaller component of the bilobular tumor

MM and TID parameter maps generally showed heterogeneity within tumors, with clear contrast in *R*, $f_{i}$, $D_{i}$, and $D_{e}$ between regions in which each model was preferred. An example is shown in Figure 7, where a central region of high $D^{\prime }$ favors the TID model. In this region, MM returns high and low *R*, low $D_{i}$ and $f_{i}$, and high $D_{e}$. Around the rim, where MM is favored, $D^{\prime }$ is lower than in the center, corresponding to lower *R* and $D_{e}$ estimates, and higher $f_{i}$ and $D_{i}$ estimates. Conventional ADC values are similar to $D^{\prime }$ in regions where TID is preferred (comparing bottom left and top right panels of Figure 7), while ADC is consistently higher than $D^{\prime }$ in regions preferred by MM. This results from the inability of a single diffusivity to describe MM regions, with $D^{\prime }$ reflecting an average of high and low diffusivities at short and long diffusion times, respectively, along with the fact that ADC was measured at the short diffusion time only. Voxel‐wise correlations between $D^{\prime }$ and ADC are shown in Supporting Information Figure S5, illustrating the tendency for ADC to be higher than $D^{\prime }$ when both parameters are low, but similar to $D^{\prime }$ when both parameters are high.

#### 3.4.4 Microstructural parameters are sensitive to radiotherapy‐induced changes

**Figure 8:**
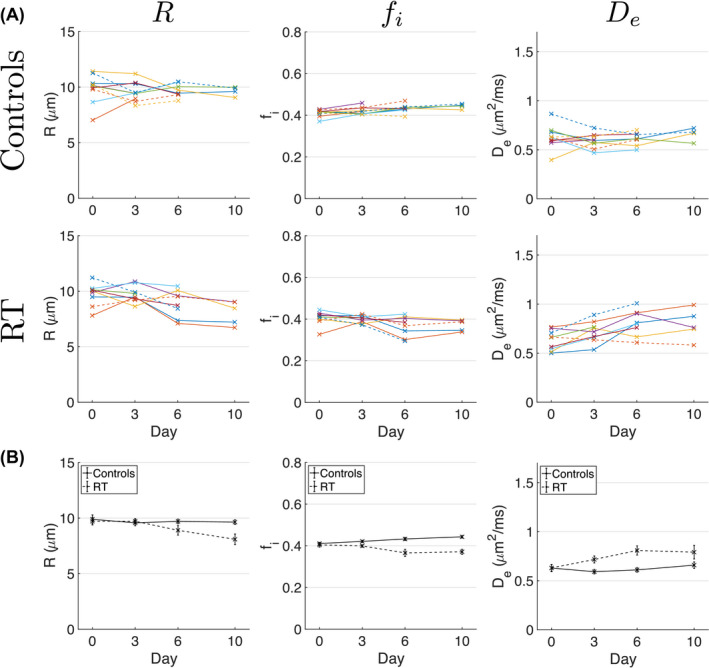
MM parameters over time. A, Median values for MM parameters, from regions where MM is favored over TID, plotted as a function of time for control (first row) and radiotherapy (RT, second row) groups. Individual data points are median values for a given tumor, and lines connect the same tumor at different time points. B, Same data as (A), with each parameter plotted as mean ± standard error over subjects, for control (solid lines) and treated (dashed lines) groups

Figure 8 plots median MM parameters, from voxels where MM was preferred over TID, as a function of time. At day 0, mean ± SD values (over all tumors) of median MM parameters were *R* = 10 ± 1 μm, $f_{i}\;=\;0.41\;\pm \;0.03$, and $D_{e}\;=\;0.6\;\pm \;0.1\;\mu m^{2}$/ms. Comparing parameter medians between groups showed a non‐significant difference in *R* (*P* = .064), while $f_{i}$ and $D_{e}$ were significantly different (*P* < .0001 and *P* = .0002). There were slight increases in $f_{i}$ in the controls and decreases in the treated group, while $D_{e}$ tended to increase in the treated group.

## 4 DISCUSSION

Biomarkers derived from DW‐MRI, including ADC and parameters from microstructural models, are being investigated as potential markers of treatment response in oncology.^8^, ^9^, ^11^, ^12^, ^26^ ADC is relatively straightforward to measure and has been shown to have sensitivity to therapy‐induced changes. However, it lacks specificity as it can be influenced by various cellular‐level features, without being able to characterize these features directly. This has motivated the use of more complex models, which potentially yield more specific biomarkers and may help resolve ambiguities in conventional ADC measurements.^11^, ^27^, ^28^ For any model‐derived biomarker, understanding the spatial and temporal validity of the model is an important part of biomarker validation. The present work's evaluation of model suitability provides two insights into tumor microstructure and treatment response. First, there is spatial and radiotherapy‐related variation in different models’ suitability for describing water diffusion in tumor tissue, potentially reflecting different and changing microenvironments. Second, within restricted diffusion regions, microstructural parameters are sensitive to radiotherapy‐induced changes, and potentially provide more specific microstructural information than conventional DW‐MRI‐derived biomarkers. Finally, the work provides important information on the technical and biological validity of the proposed %MM biomarker.

The observed increase in conventional ADC in the treated group is consistent with the majority of preclinical studies investigating ADC in response to single‐fraction radiotherapy, with increases reported in models of fibrosarcoma,^29^ glioma,^12^, ^30^ non‐Hodgkin's lymphoma,^31^ and colorectal cancer.^16^ Such increases have been hypothesized to reflect decreases in cellularity,^29^, ^30^ and have been associated with histologically observed apoptosis.^31^ A previous study investigating the same cell line and mouse strain as that used in the present work reported an initial *decrease* in ADC less than one day following 10 Gy radiotherapy given in 2 fractions^32^; the present work would not capture such a change given its first time point of 3 days. However, even at time points up to 7 days post‐treatment, ADC in treated tumors tended to be similar to or lower than baseline values,^32^ in contrast to the increases observed in the present study. This difference may be due to the exclusion of necrotic regions in the study of Zhang et al,^32^ with the present study including such regions in ADC analyses. If these regions are excluded, by calculating median ADC values from only those voxels favored by MM, changes relative to baseline in the treated group are reduced. For example, when all tumor voxels are included, group‐mean ADC in the treated group increases by 11%, 23%, and 26% at days 3, 6, and 10, relative to baseline; when including only MM‐favored voxels, these values drop to 8%, 9% and 5%, respectively. ADC differences between control and treated groups are also reduced when including only MM‐favored voxels. For example, when all tumor voxels are included, there is an 18% difference in group‐mean ADCs between controls and treated at day 6; when including only MM‐favored voxels, this drops to 7%. Such effects highlight how the link between ROI definition and tumor heterogeneity can affect analyses. In the present study, whole‐tumor ADC was found to correlate with % necrosis from histology, while Zhang et al observed correlations between ADC in viable regions and apoptotic markers,^32^ suggesting that ADC measurements are sensitive to multiple forms of cell death.

By directly modelling cellular‐level features, microstructural models have the potential to provide a more specific interpretation of changes in the DW‐MRI signal. The microstructural modelling in the present study suggests that radiotherapy results in a decrease in intracellular signal fraction, and an increase in extracellular diffusivity, implying that these are the underlying microstructural changes which lead to the observed increase in ADC. Although these microstructural changes cannot be quantitatively validated with the present data, a decrease in $f_{i}$ is consistent with a loss of cells due to radiotherapy‐induced cell death, and is consistent with DW‐MRI measurements in 9L gliomas treated with radiotherapy.^12^ It should also be noted that the absolute intracellular signal fractions in the present study (∼0.4) were higher than those estimated in gliomas (∼0.1^12^), qualitatively consistent with separate reports of relatively high extracellular spaces in gliomas.^33^, ^34^ However, the values reported in the present study are lower than those reported for other colorectal cancer models using similar DW‐MRI methods (∼0.68‐0.84^11^). The changes and heterogeneity in $D_{e}$ suggest that it is neither a static nor uniform parameter, as implicitly assumed when fixing it to a single value in fitting routines. Although further work is needed to understand its relationship with tumor microstructure, $D_{e}$ may itself be a useful biomarker, with the observed increase suggesting that radiotherapy has an influence on the extracellular space. Single and fractionated radiotherapy doses have previously been shown to affect the extracellular matrix, causing a reduction in collagen matrix stiffness.^35^ This reduction in stiffness, however, was not associated with a change in the collagen architecture, a microstructural property that $D_{e}$ may be hypothesized to have sensitivity to. Validation of $D_{e}$ as a biomarker therefore requires further experiments, in which the extracellular matrix is modulated in a controlled way.

Taking the ADC and model comparison data together, shows consistency between the gradual ADC increase in the controls and the gradual decrease in %MM, suggesting that tumor growth due to the lack of treatment is accompanied by necrosis. In non‐necrotic regions, there is a trend for increasing $f_{i}$ and little change in *R*, consistent with cell density increasing as tumors grow. The larger ADC increase in the treated group is consistent with the larger %MM decrease, with the $f_{i}$ and $D_{e}$ changes suggesting that radiotherapy affects properties of the non‐necrotic regions, as well as changing the proportion of necrotic tissue.

As shown with the fitting simulations, the increased specificity offered by microstructural models has associated drawbacks in terms of model parameter accuracy and precision, and the need to fix parameters is a clear limitation of the approach. The present work's approach of using a range of fixed $D_{i}$ values was chosen as a compromise between fitting it directly, which results in unstable fits, and fixing it to a single a priori value for all voxels at all time points, which may bias other parameter estimates. Even with this approach, $D_{i}$ tends to be estimated poorly, showing the difficulty in robustly characterizing diffusion within cells. The lack of sensitivity to intracellular diffusion with PGSE acquisitions has been demonstrated previously,^36^ and may be overcome using oscillating gradient sequences to reach shorter diffusion times.^12^ More generally, parameter degeneracy is a recognized problem with this type of model,^37^ and future work could investigate if tumor microstructural estimates can be improved with alternative acquisition strategies, following examples in white matter models.^38^, ^39^

Using model comparison to identify sub‐regions shares similarities with previous efforts to characterize intra‐tumor heterogeneity using clustering^29^, ^40^, ^41^ or probabilistic classification^42^ of multi‐contrast MR data. While it is beyond the scope of the present work to compare model comparison and multi‐contrast approaches to identifying sub‐regions, the inclusion of additional data beyond DWI would be expected to aid the characterization. For example, including $T_{2}$ alongside time‐dependent diffusion measurements may prove useful, building on the diffusion and relaxation classification developed by Xing et al.^42^

The model comparison procedure considered in the present work also shares similarities with the approach used recently by Jiang et al,^12^ in that both utilize diffusion time dependence to distinguish between qualitatively different tumor sub‐regions. Jiang et al acquired pulsed‐gradient and oscillating‐gradient diffusion data over a range of diffusion times, and used a model selection process to determine whether a time‐independent diffusivity model or their IMPULSED (Imaging Microstructural Parameters Using Limited Spectrally Edited Diffusion) model was preferred on a voxel‐wise basis, with the aim of differentiating viable regions from late‐stage apoptotic or necrotic regions in 9L gliomas.^12^ While conceptually similar, the present work complements this approach by showing that a similar framework can be employed with a narrower range of diffusion times from only pulsed‐gradient acquisitions, and that the approach can be applied to tumors outside the brain. In addition, the present work's inclusion of day 6 and day 10 time points allows longer term post‐radiotherapy changes to be investigated, extending the 4 day range covered by Jiang et al.^12^ Perhaps most importantly, the present work not only used model comparison to determine the voxels from which to extract model parameters, but also used it to obtain a novel quantitative biomarker, %MM, whose technical and biological validity were evaluated through in silico simulations, and comparison with histology, respectively.

The in silico simulations provide important information about the technical performance of %MM estimates. The high specificity shows that true TID voxels are rarely misclassified as MM, while the lower sensitivity shows that true MM voxels have a tendency to be misclassified as TID. This is a clear limitation of the technique, hypothesized to be related to the difference in complexity of the two models being compared; with noisy data, the TID model with one parameter can appear more favorable than the MM model with four parameters, resulting in a noise‐dependent underestimation of the proposed biomarker. While the existence and magnitude of such a bias will depend on the specific nature of the models being compared, as well as the SNR of the data, we suggest that methods seeking to identify and quantify sub‐regions with model comparison should be tested with validation simulations, so that bias and precision can be evaluated; this should form part of the technical validation of derived biomarkers.^2^ The in vivo results provide evidence for the ability of model comparison techniques to identify qualitatively different tumor sub‐regions, and to assess changes in these regions in response to treatment. Specifically, the changes in %MM suggest that both groups had a reduction in the amount of tissue characterized by restricted diffusion, with a larger and earlier decrease in the treated tumors, consistent with radiotherapy‐induced cell death. Note that longitudinal changes in %MM assess the relative proportions of tumor sub‐regions over time; this methodology does not attempt to directly compare the same voxel at different time points, in contrast to approaches such as the functional diffusion map.^26^ Applying such methods to track changes in individual voxels in the current study would be challenging, due to the lack of normal anatomical structure to guide registration, and the difficulty in establishing voxel‐wise correspondence in tumors which are changing shape and size. The negative correlation between %MM and histology‐derived % necrosis provides support for the hypothesis that regions favored by TID correspond to necrotic or oedematous regions, although the histological analysis did not quantify oedema. A further limitation of the biological validation is that histology results come from only a single slice, whose location can only approximately be matched to an imaging slice. Spatial correspondence between histology and imaging is also hindered by shrinkage and distortion of histological samples due to fixation and sectioning. Improved methods for comparison of histology and imaging, such as the use of tumor‐specific moulds and image registration,^41^ would provide a more comprehensive biological validation. Taken together, the in vivo and in silico results suggest that %MM is related to tumor necrosis, although actual necrotic fractions will be lower than %MM suggests.

The model comparison provides similar information to the alternative classification using an ADC threshold, in that both approaches yield metrics that negatively correlate with % necrosis. However, as ADC values will vary depending on the acquisition protocol (eg, sequences with different diffusion times), it is unlikely that a single ${\text{ADC}}_{\mathrm{cut}-\mathrm{off}}$ will apply across different studies, limiting the utility of this classification approach. Moreover, relying solely on ADC does not provide the more specific microstructural information that inherently comes with the %MM approach.

These results provide an initial step in the validation of %MM. Further technical and biological validation is required if it is to become a robust tool in research or clinical settings. While the technical performance here is acceptable, further work is needed to understand how the acquisition protocol affects sensitivity and specificity; this is especially important for clinical applications where gradient strength will typically be lower. The impact of diffusion time should also be explored, given its critical role in distinguishing time‐dependent and time‐independent diffusion regimes. As with all biomarkers seeking to detect treatment‐induced changes, the magnitude of expected biological changes needs to be compared with parameter accuracy and precision.^28^ Further biological validation would involve other tumor types and interventions, along with improved histology‐imaging comparisons.

## 5 CONCLUSIONS

The diffusion model comparison presented here provides two insights into tumor microstructure and treatment response. First, there is spatial and radiotherapy‐related variation in different models’ suitability for describing water diffusion in tumor tissue, potentially reflecting different and changing microenvironments. Second, within restricted diffusion regions, microstructural parameters are sensitive to radiotherapy‐induced changes, and potentially provide more specific microstructural information than conventional DW‐MRI‐derived biomarkers.

These results suggest that tumor heterogeneity should be considered when applying models to pre‐treatment and post‐treatment DW‐MRI data. More generally, models describing any quantitative imaging data may need to account for spatial and treatment‐related changes in model suitability. This ensures appropriate use of models and potentially yields novel biomarkers of treatment response based on physiological differences between tumor sub‐regions. The biological and technical validation of the proposed %MM biomarker suggests it correlates with, but, due to the effects of noise, overestimates, % necrosis.

## CONFLICT OF INTEREST

G.J.M. Parker has a shareholding and part time appointment and directorship at Bioxydyn Ltd. which provides diffusion MRI services. J.H. Naish has a part time secondment at Bioxydyn Ltd.

## Supporting information


**FIGURE S1** Model selection procedure with *discrete*‐ $D_{i}$ fitting. The microstructural model (MM) was fitted voxel‐wise to signals normalized to $b\;=\;0\;s/{\mathrm{mm}}^{2}$, with separate fits for $D_{i}$ fixed to $0.5,\;1.0,\;1.5,\;2.0,\;2.5\;\mu m^{2}$/ms. Fits for one example voxel, A (red box on $b\;=\;0\;s/{\mathrm{mm}}^{2}$ image), within the tumor ROI (black outline) are shown. The fit with the highest $R^{2}$ was accepted, and was then compared with the time‐independent diffusion (TID) model, as described in the main text and in Figure 1
**FIGURE S2** Violin plots of parameter distributions from fitting simulations. A‐D, show results for ground truth $D_{i}\;=\;0.2,\;1.1,\;2.0$, and $2.9\;\mu m^{2}$/ms, respectively. In each case, distributions show results from 1500 fits for *fit*‐ $D_{i}$ and *discrete*‐ $D_{i}$, for 24 microstructures with different ground truths, identified by the black horizontal lines. For example, microstructure 1 in (A) has a ground truth of *R* = 5 μm, $D_{i}\;=\;0.2\;\mu m^{2}$/ms, $D_{e}\;=\;0.2\;\mu m^{2}$/ms, and $f_{i}\;=\;0.25$. The red square represents the median of each distribution. For example, a parameter estimated with high accuracy and precision would have a narrow gray band and red square both centered on the black line
**FIGURE S3** Median conventional ADC and % necrosis. Whole‐tumor median ADC is plotted against percentage necrosis for control (circles) and radiotherapy (RT, crosses) groups. Using all data points, there is a significant positive correlation (Pearson's correlation coefficient, *ρ* = 0.56, *P* = .016)
**FIGURE S4** Conventional ADC threshold and % necrosis. A, A range of ADC thresholds, ${\text{ADC}}_{\mathrm{thresh}}$ = 0.1‐3 $\mu m^{2}$/ms, were applied to all central‐slice conventional ADC datasets; for each threshold and dataset, the percentage of voxels with ADC below the given threshold was calculated. Curves show this percentage as a function of threshold for all tumors at all time points; for example, no voxels have $\text{ADC}\;<\;0.1\;\mu m^{2}$/ms, and all voxels have $\text{ADC}\;\leq \;3\;\mu m^{2}$/ms. B, For each threshold, the corresponding percentage of voxels below the threshold was correlated with % necrosis from histology; this only used ADC data from subjects’ final scan. Pearson's correlation coefficient, *ρ*, is plotted as a function of ${\text{ADC}}_{\mathrm{thresh}}$, showing that the maximum absolute *ρ* is obtained with a threshold of $1.07\;\mu m^{2}$/ms, termed the ${\text{ADC}}_{\mathrm{cut}-\mathrm{off}}$. C, The correlation for this maximum absolute *ρ* is shown, for control (circles) and radiotherapy (RT, crosses) groups (*ρ* = −0.65, *P* = .003)
**FIGURE S5** Bivariate histograms of whole‐tumor voxel‐wise $D^{\prime }$ and ADC values, for control (top) and radiotherapy‐treated (bottom) tumors. In each case, plots are shown for all subjects (columns) at all time points (rows). The black line in each plot represents $D^{\prime }\;=\;ADC$; the colour scale represents the normalized bin count and is the same for each plot. When both parameters are low, the tendency is for ADC to be higher than $D^{\prime }$ (points are above the black line), while when both parameters are high, ADC and $D^{\prime }$ are similar (points lie closer to the black line). This trend is expected as $D^{\prime }$ is obtained from short and long diffusion times, and reflects an average of high and low diffusivities when diffusion is time dependent, while ADC is measured only at the short diffusion time where the diffusivity will be higher; the two parameters are equivalent when diffusion is time‐independent, which here tends to be at higher diffusivities. Note that these plots include $D^{\prime }$ values from all tumor voxels, including those where the MM model is preferred over the TID model, that is, where diffusion is time dependent
