# Supplementary material for: Diffusion model comparison identifies distinct tumor sub‐regions and tracks treatment response
Source: Magn Reson Med. 2020 Feb 14;84(3):1250–63. doi: 10.1002/mrm.28196 (PMC7317874; doi:10.1002/mrm.28196)
Supplement: Supplementary file 1 — FIGURE S1 Model selection procedure with discrete‐ Di fitting. The microstructural model (MM) was fitted voxel‐wise to signals normalized to b=0s/mm2, with separate fits for Di fixed to 0.5,1.0,1.5,2.0,2.5μm2/ms. Fits for one example voxel, A (red box on b=0s/mm2 image), within the tumor ROI (black outline) are shown. The fit with the highest R2 was accepted, and was then compared with the time‐independent diffusion (TID) model, as described in the main text and in Figure 1 FIGURE S2 Violin plots of parameter distributions from fitting simulations. A‐D, show results for ground truth Di=0.2,1.1,2.0, and 2.9μm2/ms, respectively. In each case, distributions show results from 1500 fits for fit‐ Di and discrete‐ Di, for 24 microstructures with different ground truths, identified by the black horizontal lines. For example, microstructure 1 in (A) has a ground truth of R = 5 μm, Di=0.2μm2/ms, De=0.2μm2/ms, and fi=0.25. The red square represents the median of each distribution. For example, a parameter estimated with high accuracy and precision would have a narrow gray band and red square both centered on the black line FIGURE S3 Median conventional ADC and % necrosis. Whole‐tumor median ADC is plotted against percentage necrosis for control (circles) and radiotherapy (RT, crosses) groups. Using all data points, there is a significant positive correlation (Pearson's correlation coefficient, ρ = 0.56, P = .016) FIGURE S4 Conventional ADC threshold and % necrosis. A, A range of ADC thresholds, ADCthresh = 0.1‐3 μm2/ms, were applied to all central‐slice conventional ADC datasets; for each threshold and dataset, the percentage of voxels with ADC below the given threshold was calculated. Curves show this percentage as a function of threshold for all tumors at all time points; for example, no voxels have ADC<0.1μm2/ms, and all voxels have ADC≤3μm2/ms. B, For each threshold, the corresponding percentage of voxels below the threshold was correlated with % necrosis from histology; t [file MRM-84-1250-s001.pdf]

Supporting information

discrete-D_i fitting

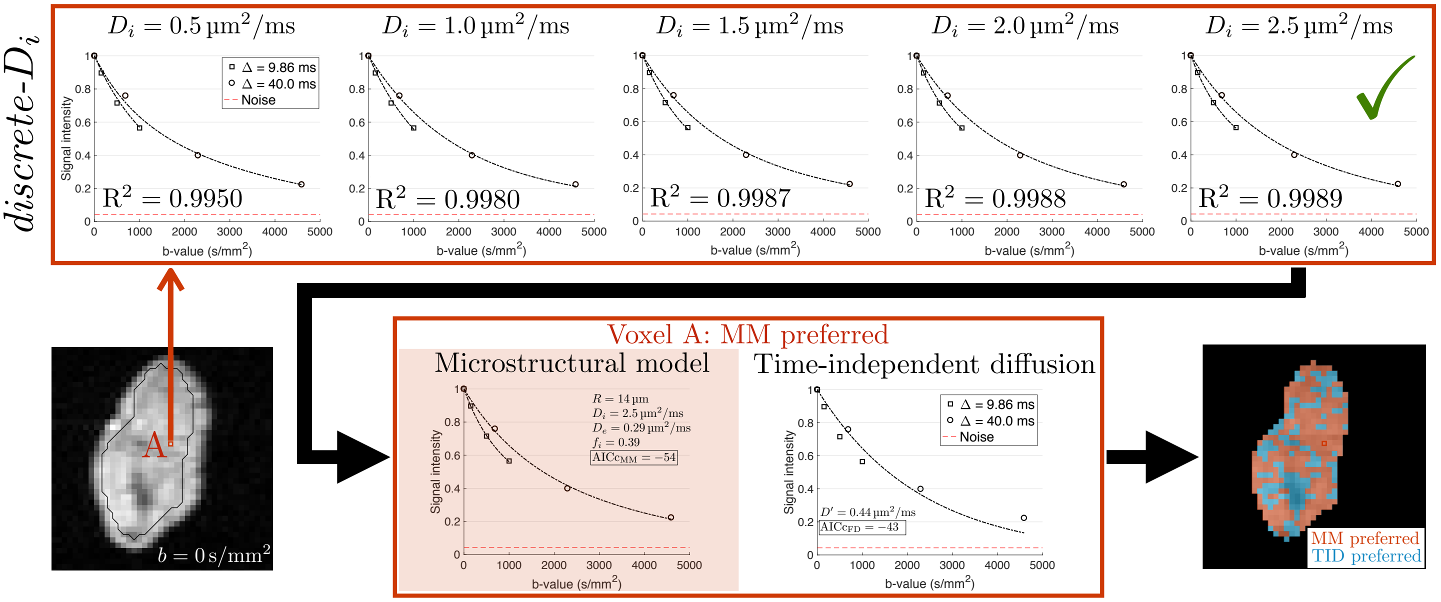

FIG. S1. Model selection procedure with *discrete-*D_i fitting. The microstructural model (MM) was fitted voxel-wise to signals normalised to $b = 0\,s/mm^2$, with separate fits for D_i fixed to $0.5, 1.0, 1.5, 2.0, 2.5\,\mu m^2/ms$. Fits for one example voxel, A (red box on $b = 0\,s/mm^2$ image), within the tumour ROI (black outline) are shown. The fit with the highest R^2 was accepted, and was then compared with the time-independent diffusion (TID) model, as described in the main text and in Figure 1.

Fitting simulations - parameter distributions

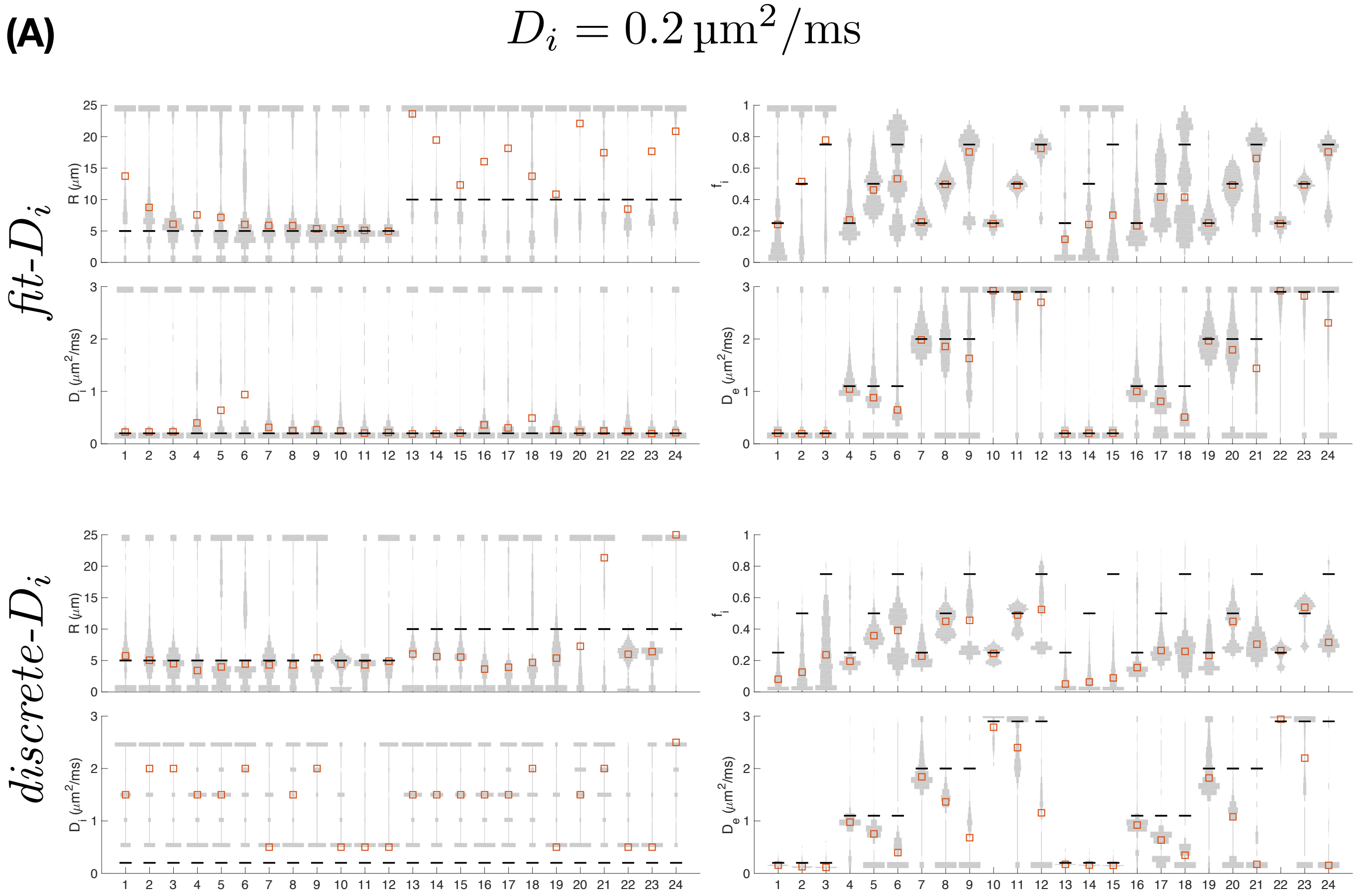

(B)

$$D_i = 1.1\,\mu\mathrm{m}^2/\mathrm{ms}$$

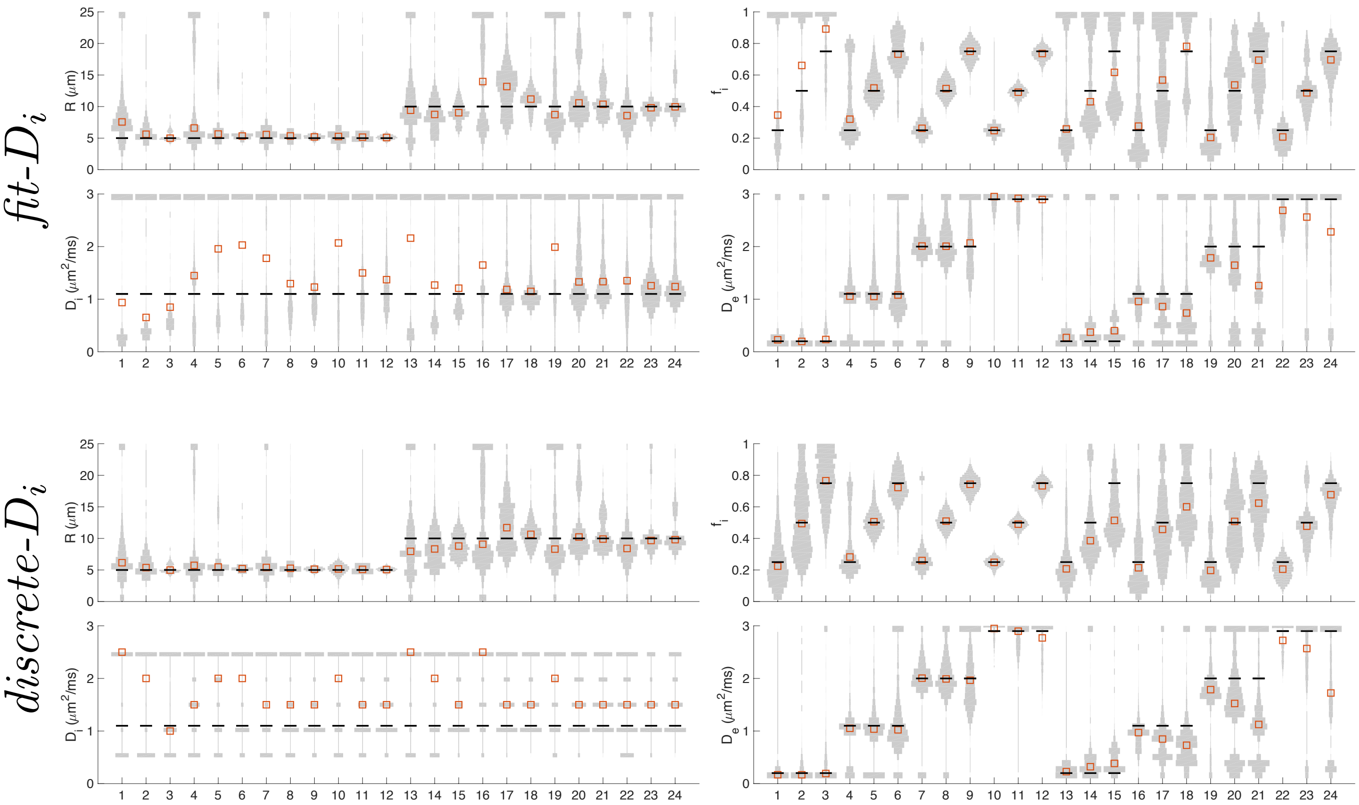

(C) $D_i = 2.0\,\mathrm{\mu m^2/ms}$

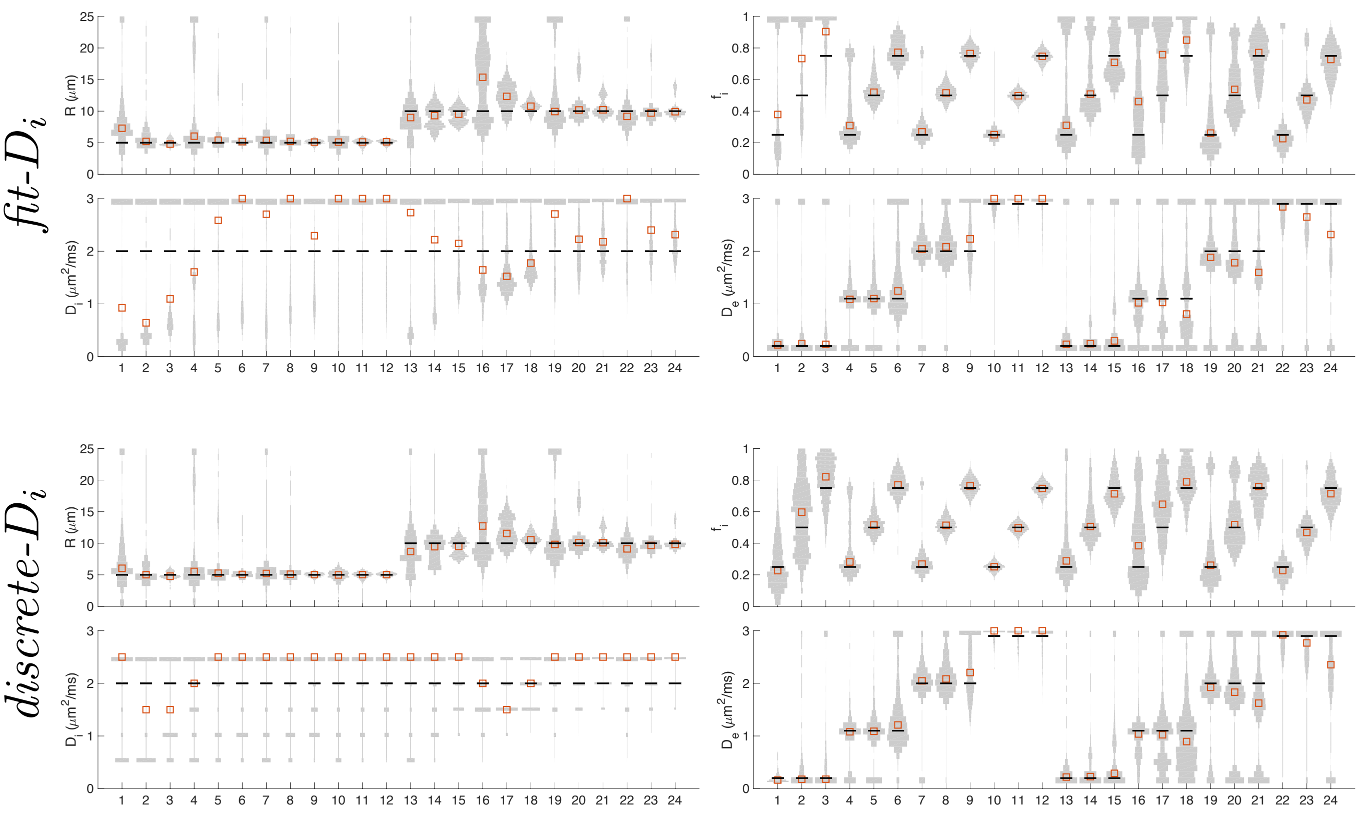

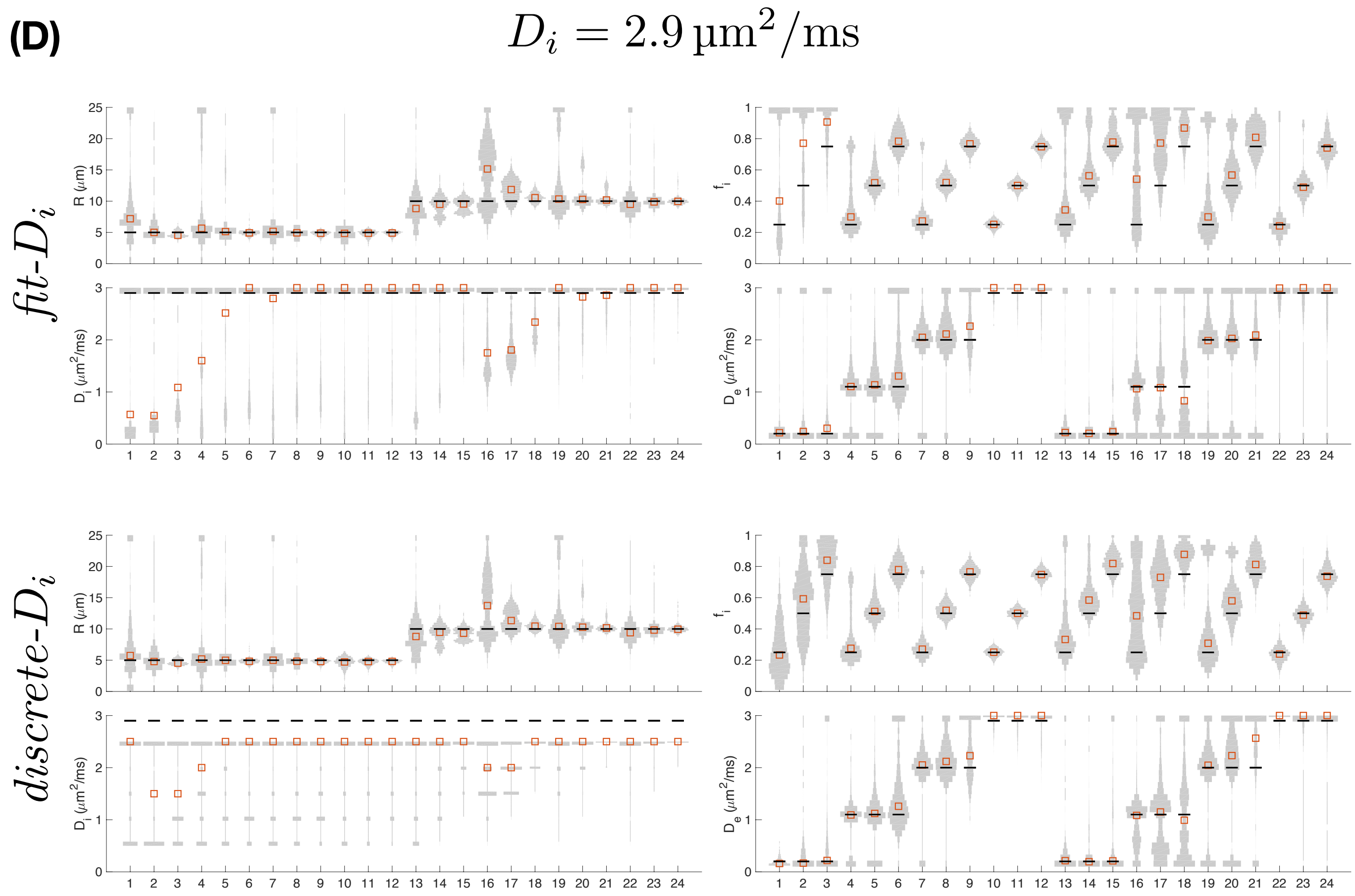

FIG. S2. Violin plots of parameter distributions from fitting simulations. **(A)** - **(D)** show results for ground truth $D_i = 0.2, 1.1, 2.0$, and $2.9\,\mu m^2/ms$, respectively. In each case, distributions show results from 1500 fits for *fit*-D_i and *discrete*-D_i, for 24 microstructures with different ground truths, identified by the black horizontal lines. For example, microstructure 1 in **(A)** has a ground truth of $R = 5\,\mu m$, $D_i = 0.2\,\mu m^2/ms$, $D_e = 0.2\,\mu m^2/ms$, and $f_i = 0.25$. The red square represents the median of each distribution. For example, a parameter estimated with high accuracy and precision would have a narrow grey band and red square both centred on the black line.

Conventional ADC relates to histology measurement of necrosis

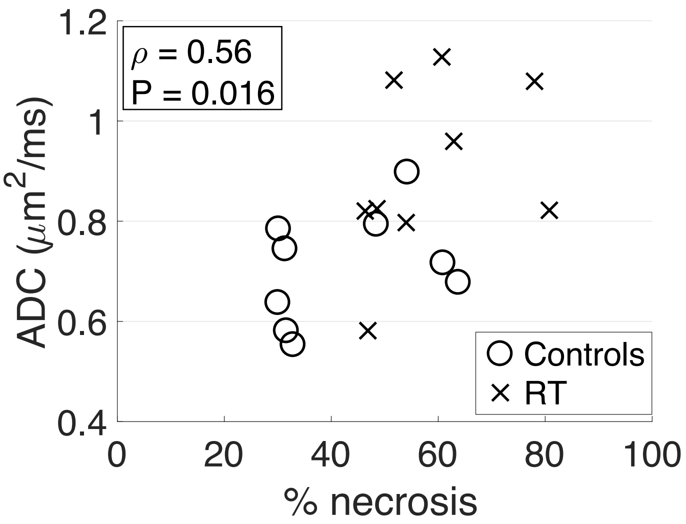

FIG. S3. Median conventional ADC and %necrosis. Whole-tumour median ADC plotted against percentage necrosis for control (circles) and radiotherapy (RT, crosses) groups. Using all data points, there is a significant positive correlation (Pearson's correlation coefficient, $\rho = 0.56, P = 0.016$).

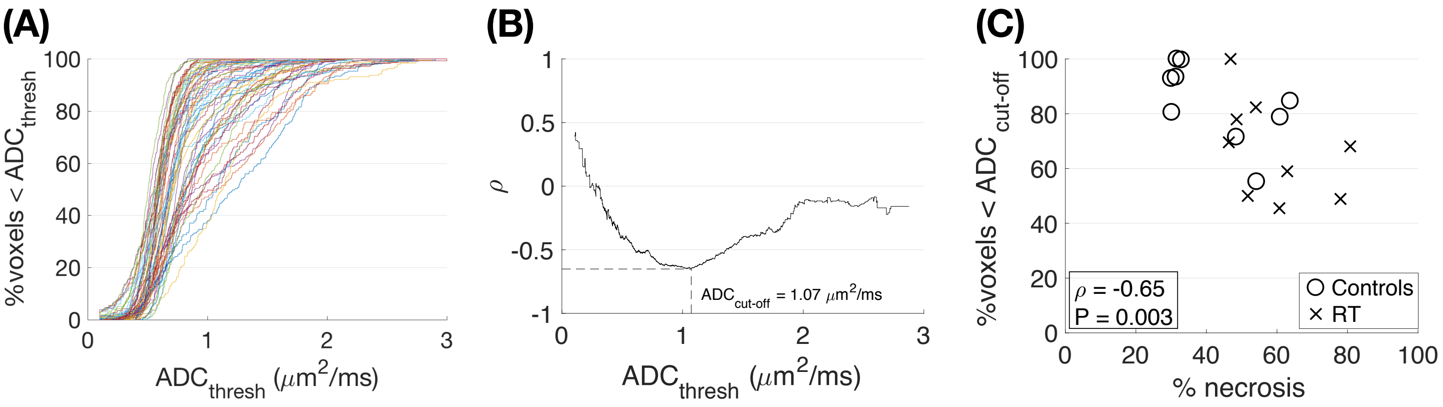

FIG. S4. Conventional ADC threshold and %necrosis. **(A)** A range of ADC thresholds, $\mathrm{ADC_{thresh}} = 0.1 - 3\,\mu\mathrm{m}^2/\mathrm{ms}$, were applied to all central-slice conventional ADC datasets; for each threshold and dataset, the percentage of voxels with ADC below the given threshold was calculated. Curves show this percentage as a function of threshold for all tumours at all time points; e.g. no voxels have ADC $< 0.1\,\mu\mathrm{m}^2/\mathrm{ms}$, and all voxels have ADC $\leq 3\,\mu\mathrm{m}^2/\mathrm{ms}$. **(B)** For each threshold, the corresponding percentage of voxels below the threshold was correlated with %necrosis from histology; this only used ADC data from subjects' final scan. Pearson's correlation coefficient, ρ, is plotted as a function of $\mathrm{ADC_{thresh}}$, showing that the maximum absolute ρ is obtained with a threshold of $1.07\,\mu\mathrm{m}^2/\mathrm{ms}$, termed the $\mathrm{ADC_{cut-off}}$. **(C)** The correlation for this maximum absolute ρ is shown, for control (circles) and radiotherapy (RT, crosses) groups ($\rho = -0.65, P = 0.003$).

D' - ADC correlation

Controls

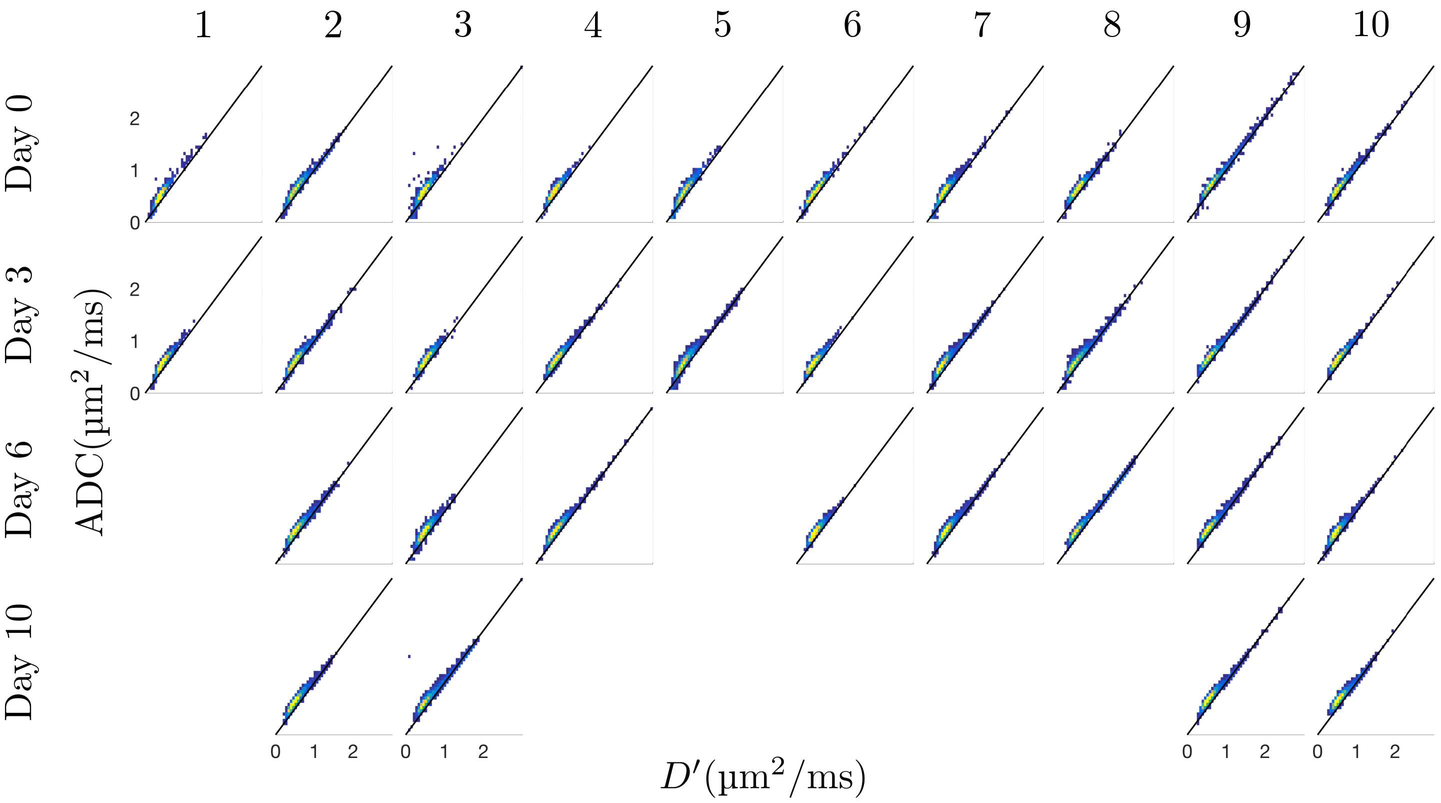

Radiotherapy

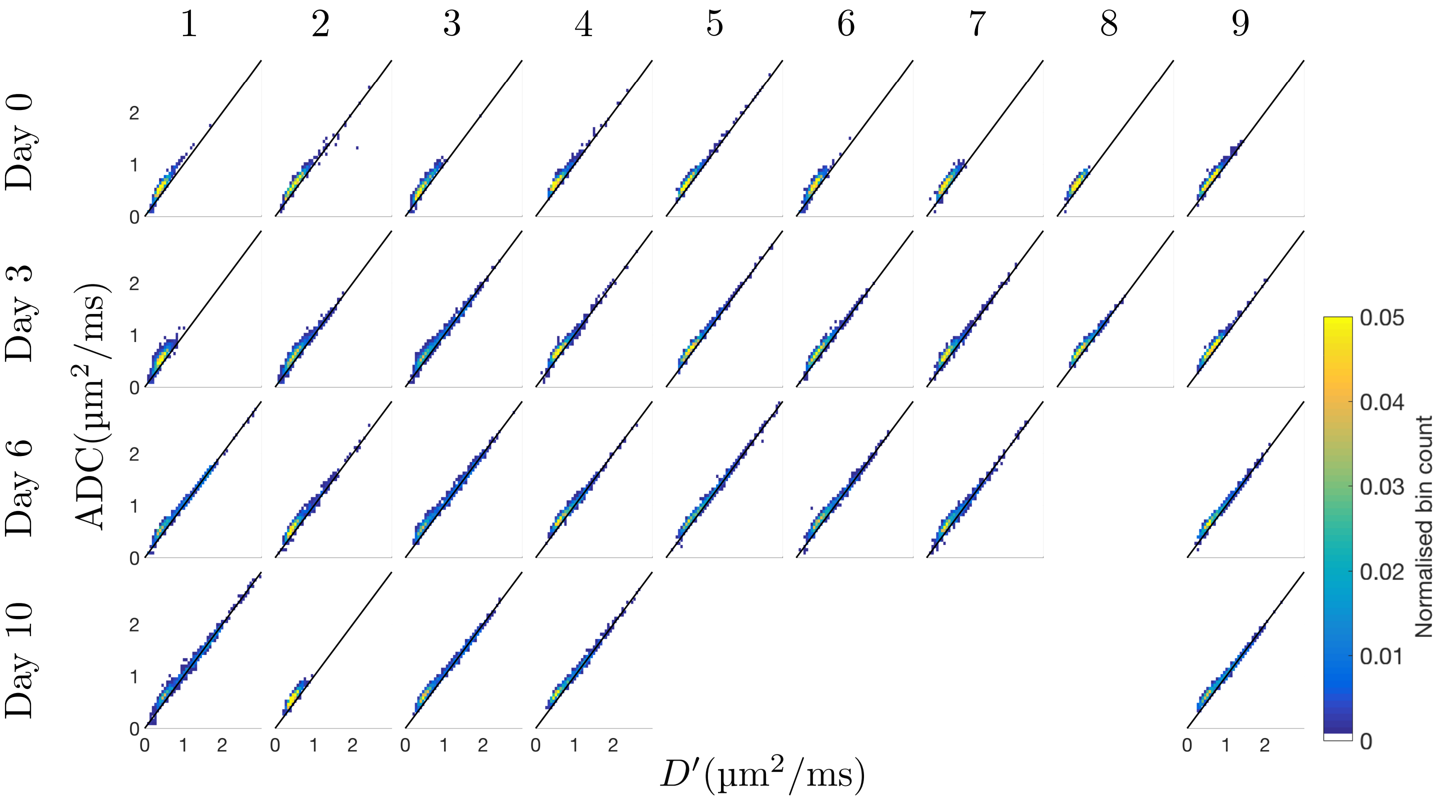

FIG. S5. Bivariate histograms of whole-tumour voxel-wise D' and ADC values, for control (top) and radiotherapy-treated (bottom) tumours. In each case, plots are shown for all subjects (columns) at all time points (rows). The black line in each plot represents $D' = ADC$; the colour scale represents the normalised bin count and is the same for each plot. When both parameters are low, the tendency is for ADC to be higher than D' (points are above the black line), while when both parameters are high, ADC and D' are similar (points lie closer to the black line). This trend is expected as D' is obtained from short and long diffusion times, and reflects an average of high and low diffusivities when diffusion is time-dependent, while ADC is measured only at the short diffusion time where the diffusivity will be higher; the two parameters are equivalent when diffusion is time-independent, which here tends to be at higher diffusivities. Note that these plots include D' values from all tumour voxels, including those where the MM model is preferred over the TID model, that is, where diffusion is time-dependent.
